# Supplementary material for: Renal Coenzyme A (CoA) Production Fuels Stem Cell Proliferation and Tumor Growth
Source: bioRxiv. 2025 Aug 11:2025.08.08.669325. Preprint. [Version 1] doi: 10.1101/2025.08.08.669325 (PMC12363785; doi:10.1101/2025.08.08.669325)
Supplement: Supplement 1 [file media-1.pdf]

Renal Coenzyme A (CoA) Production Fuels Stem Cell Proliferation and Tumor Growth

Supplemental Table

Supplementary table1: supplier, catalog number, and concentration of vitamin used in this study
Supplementary table2: prediction of transcription factors targeting CoA pathway enzymes using TF2TG
Supplementary table3: fly genotypes
Supplementary table4: primers used in this study

Supplementary table1. Information about vitamin supplementation		
	Supplier and Catalog number	Final concentration
Vitamin B1 (thiamine)	Sigma, T4625	0.2 mM
Vitamin B2 (riboflavin)	Sigma, R4500	0.1 mM
Vitamin B3 (nicotinic acid)	Sigma, N4126	5 mM
Vitamin B5 (pantothenate acid)	Sigma, 21210	2.5 mM
Vitamin B6 (pyridoxine)	Sigma, P9755	0.5 mM
Vitamin B7 (Biotin)	Sigma, B4501	0.01 mM

Supplementary table2. Transcription factor prediction

Query gene	TF FBgn	TF Symbol	Peak Count	Motif Count	Location	Protein-Protein Interactors	Genetic Interactors
ppcs	FBgn0259750	ab	0	3	upstream		
Ppcdc	FBgn0259750	ab	0	1	upstream		
fbl	FBgn0259750	ab	0	1	upstream		
Ppat-Dpck	FBgn0000022	ac	0	2	upstream	da	sc, h (pubmed)
Ppat-Dpck	FBgn0000413	ac	0	6	upstream		
CG5828	FBgn0000022	ac	0	2	upstream	da	sc, h (pubmed)
fbl	FBgn0000022	ac	0	1	upstream		sc, ttk (pubmed)
Ppat-Dpck	FBgn0005694	Aef1	0	2	upstream		
Ppcdc	FBgn0003270	amos	0	1	upstream		da (pubmed)
ppcs	FBgn0000097	aop	0	1	upstream		pnt, ttk (pubmed)
Ppcdc	FBgn0000097	aop	0	1	upstream		H (pubmed)
CG5828	FBgn0000097	aop	0	1	upstream		H, pnt (pubmed)
Ppat-Dpck	FBgn0000137	ase	0	2	upstream	da	
CG5828	FBgn0000137	ase	0	2	upstream	da	
fbl	FBgn0000137	ase	0	1	upstream		
ppcs	FBgn0004870	bab1	0	3	upstream		
Ppcdc	FBgn0004870	bab1	0	7	upstream		
Ppat-Dpck	FBgn0004870	bab1	0	1	upstream		
Dpck	FBgn0004870	bab1	0	2	upstream		
CG5828	FBgn0004870	bab1	0	2	upstream		
fbl	FBgn0004870	bab1	0	2	upstream		
Ppcdc	FBgn0015602	BEAF-32	0	1	upstream	Dref	
CG5828	FBgn0015602	BEAF-32	0	2	upstream	Dref	
fbl	FBgn0015602	BEAF-32	0	1	upstream	Dref	
ppcs	FBgn0045759	bin	0	2	upstream		
ppcs	FBgn0045759	bin	0	2	intron		
Dpck	FBgn0045759	bin	0	1	upstream	prd, D	
CG5828	FBgn0045759	bin	1	1	upstream	D	
fbl	FBgn0045759	bin	0	4	intron	D	
fbl	FBgn0045759	bin	0	1	upstream	D	
ppcs	FBgn0035625	Blimp-1	0	8	upstream		
ppcs	FBgn0035625	Blimp-1	0	4	intron		
CG5828	FBgn0035625	Blimp-1	0	1	upstream		
ppcs	FBgn0004893	bowl	0	1	upstream		
CG5828	FBgn0004893	bowl	0	1	upstream		
ppcs	FBgn0283451	br	2	0	upstream	Rel, rib	Met (pubmed)
Ppat-Dpck	FBgn0283451	br	1	0	upstream	rib	
Dpck	FBgn0283451	br	1	0	upstream	rib	
Dpck	FBgn0000210	br	0	1	upstream		
CG5828	FBgn0283451	br	1	0	upstream	rib	
fbl	FBgn0283451	br	1	0	upstream	rib	
fbl	FBgn0000210	br	0	4	intron		
ppcs	FBgn0000210	br-PE	0	1	upstream		
ppcs	FBgn0263108	BtbVII	0	2	upstream		
ppcs	FBgn0263108	BtbVII	0	1	intron		
Dpck	FBgn0263108	BtbVII	0	1	upstream		

CG5828	FBgn0263108	BtbVII	0	1	upstream			
fbl	FBgn0263108	BtbVII	0	1	upstream			
fbl	FBgn0263108	BtbVII	0	1	intron			
Ppat-Dpck	FBgn0025679	Bteb2	0	2	upstream			
fbl	FBgn0025679	Bteb2	0	3	upstream			
Ppat-Dpck	FBgn0000286	Cf2-PA	0	1	upstream			
Dpck	FBgn0000286	Cf2-PA	0	2	upstream	bin		
fbl	FBgn0000286	Cf2-PA	0	1	upstream	bin		
ppcs	FBgn0000286	Cf2-PB	0	8	upstream	bin		
fbl	FBgn0000286	Cf2-PB	0	1	intron	bin		
ppcs	FBgn0037446	CG10267	0	2	upstream	Dll, toy		
Ppat-Dpck	FBgn0037446	CG10267	0	2	upstream	Dll		
fbl	FBgn0037446	CG10267	0	2	upstream			
Ppcdc	FBgn0034945	CG10904	0	1	upstream			
fbl	FBgn0030532	CG11071	0	1	upstream			
ppcs	FBgn0035454	CG12029	0	3	intron			
Ppcdc	FBgn0035454	CG12029	0	1	upstream			
Ppat-Dpck	FBgn0035454	CG12029	0	1	upstream			
Dpck	FBgn0035454	CG12029	0	3	upstream			
CG5828	FBgn0035454	CG12029	0	3	upstream			
fbl	FBgn0035454	CG12029	0	2	upstream			
CG5828	FBgn0029957	CG12155	0	1	upstream	Trl		
fbl	FBgn0029822	CG12236	0	1	intron			
ppcs	FBgn0037206	CG12768	0	1	intron	Mes2		
Dpck	FBgn0037206	CG12768	0	1	upstream			
Ppcdc	FBgn0035160	CG13897	0	2	upstream			
Ppat-Dpck	FBgn0035160	CG13897	0	1	upstream			
fbl	FBgn0035160	CG13897	0	1	upstream			
Ppat-Dpck	FBgn0035407	CG14962	0	3	upstream			
Dpck	FBgn0003715	CG16778	0	1	upstream			
CG5828	FBgn0003715	CG16778	0	1	upstream			
fbl	FBgn0003715	CG16778	0	2	intron			
ppcs	FBgn0037735	CG16899	0	1	upstream			
ppcs	FBgn0037735	CG16899	0	1	intron			
Dpck	FBgn0037735	CG16899	0	1	upstream			
CG5828	FBgn0037735	CG16899	0	1	upstream			
fbl	FBgn0037735	CG16899	0	3	intron			
fbl	FBgn0037735	CG16899	0	1	upstream			
Ppat-Dpck	FBgn0035144	CG17181	0	1	upstream	Aef1		
CG5828	FBgn0035144	CG17181	0	1	upstream	pnt		
ppcs	FBgn0039905	CG2052	0	3	upstream			
ppcs	FBgn0039905	CG2052	0	4	intron			
Ppcdc	FBgn0039905	CG2052	0	1	intron			
Ppat-Dpck	FBgn0039905	CG2052	0	3	upstream			
Dpck	FBgn0039905	CG2052	0	3	upstream			
CG5828	FBgn0039905	CG2052	0	11	upstream			
fbl	FBgn0039905	CG2052	0	2	upstream			
Ppat-Dpck	FBgn0034946	CG3065	0	3	upstream			

Dpck	FBgn0034946	CG3065	0	8	upstream			
CG5828	FBgn0034946	CG3065	0	4	upstream			
fbl	FBgn0034946	CG3065	0	4	upstream			
Ppcdc	FBgn0031375	CG31670	0	1	upstream		klu (pubmed)	
Dpck	FBgn0031375	CG31670	0	4	upstream		klu, Doc2 (pubmed)	
fbl	FBgn0031375	CG31670	0	2	intron			
ppcs	FBgn0052105	CG32105	0	1	upstream			
CG5828	FBgn0053980	CG33980	0	1	upstream			
Dpck	FBgn0031573	CG3407	0	1	upstream			
ppcs	FBgn0036423	CG3919	0	1	intron			
Ppat-Dpck	FBgn0036423	CG3919	0	1	upstream			
fbl	FBgn0036423	CG3919	0	2	upstream			
fbl	FBgn0036423	CG3919	0	1	intron			
CG5828	FBgn0038787	CG4360	0	1	upstream			
Ppat-Dpck	FBgn0038787	CG4360-F1-3	0	4	upstream			
ppcs	FBgn0030432	CG4404	0	1	intron			
Ppcdc	FBgn0030432	CG4404	0	2	upstream			
fbl	FBgn0030432	CG4404	0	1	upstream			
ppcs	FBgn0038766	CG4854	0	2	intron			
Ppcdc	FBgn0038766	CG4854	0	1	upstream			
Ppat-Dpck	FBgn0038766	CG4854	0	2	upstream			
CG5828	FBgn0043457	CG5180	0	1	upstream			
ppcs	FBgn0039169	CG5669	0	1	upstream			
Dpck	FBgn0039169	CG5669	0	6	upstream			
CG5828	FBgn0039169	CG5669	0	3	upstream			
fbl	FBgn0039169	CG5669	0	3	upstream			
ppcs	FBgn0032587	CG5953	0	1	upstream	knrl, sens		
Ppcdc	FBgn0032587	CG5953	0	1	upstream	sens		
Dpck	FBgn0032587	CG5953	0	2	upstream	knrl		
CG5828	FBgn0032587	CG5953	0	1	upstream			
fbl	FBgn0032587	CG5953	0	2	intron			
ppcs	FBgn0038316	CG6276	0	2	upstream			
ppcs	FBgn0038316	CG6276	0	1	intron			
Ppcdc	FBgn0038316	CG6276	0	1	upstream			
Dpck	FBgn0038316	CG6276	0	2	upstream			
CG5828	FBgn0038316	CG6276	0	2	upstream			
fbl	FBgn0038316	CG6276	0	1	upstream			
Ppat-Dpck	FBgn0036179	CG7368	0	1	upstream			
ppcs	FBgn0033616	CG7745	0	2	upstream			
ppcs	FBgn0033616	CG7745	0	1	intron			
Dpck	FBgn0033616	CG7745	0	1	upstream			
CG5828	FBgn0033616	CG7745	0	1	upstream			
Ppat-Dpck	FBgn0039740	CG7928	0	1	upstream			
ppcs	FBgn0035824	CG8281	0	1	upstream			
ppcs	FBgn0035824	CG8281	0	1	intron			
fbl	FBgn0037722	CG8319	0	2	upstream			
Ppcdc	FBgn0036900	CG8765	0	1	upstream			
CG5828	FBgn0036900	CG8765	0	1	upstream			

fbl	FBgn0036900	CG8765	0	2	intron		
ppcs	FBgn0034810	CG9895	0	2	intron		
Ppat-Dpck	FBgn0034810	CG9895	0	1	upstream		
Dpck	FBgn0034810	CG9895	0	6	upstream		
CG5828	FBgn0034810	CG9895	0	3	upstream		
fbl	FBgn0034810	CG9895	0	2	upstream		
ppcs	FBgn0086758	chinmo	0	3	intron		
Ppcdc	FBgn0086758	chinmo	0	2	upstream		
CG5828	FBgn0086758	chinmo	0	1	upstream		
fbl	FBgn0086758	chinmo	0	1	intron		
ppcs	FBgn0000370	Crc	0	3	upstream	EcR	EcR (pubmed)
ppcs	FBgn0036126	Crc	0	2	upstream	Xrp1, crc	
Ppat-Dpck	FBgn0000370	Crc	0	1	upstream	EcR	EcR (pubmed)
ppcs	FBgn0014143	croc	0	1	upstream	opa	
ppcs	FBgn0014143	croc	0	1	intron	opa	
Ppcdc	FBgn0014143	croc	0	2	upstream		
Ppat-Dpck	FBgn0014143	croc	0	1	upstream		
Dpck	FBgn0014143	croc	0	1	upstream	prd	
fbl	FBgn0014143	croc	0	1	upstream		
ppcs	FBgn0020309	crol-F7-16	0	1	upstream		
CG5828	FBgn0020309	crol-F7-16	0	1	upstream		
CG5828	FBgn0001994	crp	0	1	upstream		
ppcs	FBgn0023094	cyc	1	0	intron		
Ppcdc	FBgn0023094	cyc	1	0	upstream		
Ppat-Dpck	FBgn0023094	cyc	1	0	upstream		
Dpck	FBgn0023094	cyc	3	0	upstream		
CG5828	FBgn0023094	cyc	2	0	upstream		
fbl	FBgn0023094	cyc	4	0	intron		
Ppcdc	FBgn0000411	D	3	0	upstream	vnd	
Ppat-Dpck	FBgn0000411	D	3	0	upstream		
Dpck	FBgn0000411	D	2	0	upstream	Doc1, bin	
CG5828	FBgn0000411	D	2	0	upstream	bin	
fbl	FBgn0000411	D	1	0	upstream	vnd, ftz-f1, ttk, bin	
ppcs	FBgn0022935	D19A	0	2	upstream		
Dpck	FBgn0022935	D19A	0	1	upstream		
CG5828	FBgn0022935	D19A	0	1	upstream		
fbl	FBgn0022935	D19A	0	1	upstream		
fbl	FBgn0022935	D19A	0	1	intron		
ppcs	FBgn0022699	D19B-F10-12	0	1	intron		
ppcs	FBgn0267821	da	1	0	upstream	ey, Fer3	
ppcs	FBgn0000413	da	0	1	upstream		
ppcs	FBgn0000413	da	0	4	intron		
Ppcdc	FBgn0267821	da	1	0	upstream	dimm, ey, Fer3	amos (pubmed)
Ppat-Dpck	FBgn0267821	da	3	0	upstream	ac, sc, l(1)sc, ase, HLH54F, ey, Fer3	sc, l(1)sc (pubmed)
CG5828	FBgn0000413	da	0	4	upstream		
CG5828	FBgn0267821	da	2	0	upstream	ac, sc, l(1)sc, ase, Fer3	sc, l(1)sc (pubmed)
Ppcdc	FBgn0000413	dei	0	5	upstream		
Ppcdc	FBgn0008649	dei	0	2	upstream		

Ppcdc	FBgn0023091	dimm	0	1	upstream	da, sqz	
Dpck	FBgn0000413	dimm	0	1	upstream		
Dpck	FBgn0023091	dimm	0	2	upstream	sqz	
ppcs	FBgn0040465	Dip3	0	1	upstream	CG4854	
Ppcdc	FBgn0040465	Dip3	0	1	intron	dl, CG4854	
Ppat-Dpck	FBgn0040465	Dip3	0	2	upstream	CG4854	
Dpck	FBgn0040465	Dip3	0	2	upstream		
CG5828	FBgn0040465	Dip3	0	2	upstream		
Ppcdc	FBgn0000462	dl	0	2	upstream		
ppcs	FBgn0000157	Dll	1	0	upstream	Dref	
Ppat-Dpck	FBgn0000157	Dll	2	0	upstream	Dref	
CG5828	FBgn0000157	Dll	1	0	upstream	Dref	
Dpck	FBgn0028789	Doc1	0	1	intron	Doc2, D	Doc2 (pubmed)
Dpck	FBgn0035956	Doc2	0	1	intron	Doc1	Doc1 (pubmed)
Dpck	FBgn0035954	Doc3	0	1	intron		
Ppat-Dpck	FBgn0010109	dpn	0	2	upstream	h, Hey	
ppcs	FBgn0000492	Dr	0	1	upstream		
ppcs	FBgn0015664	Dref	0	2	upstream	Dll	
ppcs	FBgn0015664	Dref	0	1	intron	Dll	
Ppcdc	FBgn0015664	Dref	0	1	upstream	BEAF-32	
Ppat-Dpck	FBgn0015664	Dref	0	3	upstream	Dll	
CG5828	FBgn0015664	Dref	0	2	upstream	BEAF-32, Dll	
fbl	FBgn0015664	Dref	0	1	upstream	BEAF-32	
ppcs	FBgn0015381	dsf	0	1	upstream		
Ppat-Dpck	FBgn0000504	dsx-F	0	1	upstream	ey	
CG5828	FBgn0000504	dsx-F	0	1	upstream		
ppcs	FBgn0039411	dys	0	4	upstream	tgo	
Dpck	FBgn0039411	dys	0	1	upstream	tgo	
CG5828	FBgn0015014	dys	0	1	upstream		
CG5828	FBgn0039411	dys	0	2	upstream		
Ppcdc	FBgn0000591	E(spl)	0	1	upstream	da, h	Dl, H (pubmed)
ppcs	FBgn0000546	EcR	0	1	upstream	usp, Met, tai, crc	Hr39, crc (pubmed)
ppcs	FBgn0000546	EcR	0	1	intron	usp, Met, tai, crc	Hr39, crc (pubmed)
Ppat-Dpck	FBgn0000546	EcR	2	0	upstream	usp, crc	crc (pubmed)
ppcs	FBgn0000560	eg	0	2	upstream		
ppcs	FBgn0000568	Eip75B	0	2	intron	Hr51	Kr (pubmed)
Dpck	FBgn0000568	Eip75B	0	1	upstream		
fbl	FBgn0000568	Eip75B	0	2	upstream	Hr51	
ppcs	FBgn0004865	Eip78C	0	1	upstream		
ppcs	FBgn0004865	Eip78C	0	1	intron		
ppcs	FBgn0013948	Eip93F	0	3	upstream		
ppcs	FBgn0013948	Eip93F	0	1	intron		
Dpck	FBgn0013948	Eip93F	0	1	upstream		
CG5828	FBgn0013948	Eip93F	0	2	upstream		
ppcs	FBgn0035849	ERR	0	1	intron		
Ppat-Dpck	FBgn0001981	esg-F3-5	0	2	upstream		ase (pubmed)
CG5828	FBgn0001981	esg-F3-5	0	3	upstream	Sp1	ase (pubmed)
fbl	FBgn0001981	esg-F3-5	0	2	upstream	Sp1	ase (pubmed)

ppcs	FBgn0000591	Espl	0	2	intron	da		
Ppat-Dpck	FBgn0000591	Espl	0	3	upstream	da, dpn, h		l(1)sc, H (pubmed)
Dpck	FBgn0000591	Espl	0	1	upstream	h		H (pubmed)
fbl	FBgn0000591	Espl	0	2	intron			l(1)sc (pubmed)
ppcs	FBgn0005660	Ets21c	0	2	upstream			
Ppcdc	FBgn0005660	Ets21c	0	1	upstream			
CG5828	FBgn0005660	Ets21c	0	2	upstream			
ppcs	FBgn0005658	Ets65A	0	1	upstream			
Ppcdc	FBgn0005658	Ets65A	0	1	upstream			
CG5828	FBgn0005658	Ets65A	0	1	upstream			
ppcs	FBgn0039225	Ets96B	0	1	upstream			
CG5828	FBgn0039225	Ets96B	0	1	upstream			
ppcs	FBgn0004510	Ets97D	0	2	upstream	Myc		
CG5828	FBgn0004510	Ets97D	0	2	upstream	Myc		
fbl	FBgn0005659	Ets98B	0	1	upstream			
ppcs	FBgn0005558	ey	0	3	intron	da		toy (pubmed)
Ppcdc	FBgn0005558	ey	0	1	upstream	da, hth		toy (pubmed)
Ppat-Dpck	FBgn0005558	ey	0	2	upstream	da, hth		
fbl	FBgn0005558	ey	0	1	upstream			
ppcs	FBgn0037475	Fer1	0	2	intron			
Ppcdc	FBgn0037475	Fer1	0	2	upstream			
CG5828	FBgn0037475	Fer1	0	2	upstream			
ppcs	FBgn0038402	Fer2	0	1	upstream			
CG5828	FBgn0038402	Fer2	0	2	upstream			
ppcs	FBgn0037937	Fer3	1	0	upstream	da		
Ppcdc	FBgn0037937	Fer3	0	2	upstream	da		
Ppat-Dpck	FBgn0037937	Fer3	2	0	upstream	da		
CG5828	FBgn0037937	Fer3	1	0	upstream	da		
ppcs	FBgn0000659	fkh	0	1	upstream			
ppcs	FBgn0000659	fkh	0	1	intron			
Dpck	FBgn0000659	fkh	0	3	upstream			
fbl	FBgn0000659	fkh	0	2	intron			
fbl	FBgn0001078	ftz-f1	0	1	upstream	D		
ppcs	FBgn0032223	GATAd	0	2	upstream			
ppcs	FBgn0032223	GATAd	0	1	intron			
Ppat-Dpck	FBgn0032223	GATAd	0	1	upstream			
CG5828	FBgn0032223	GATAd	0	1	upstream			
fbl	FBgn0032223	GATAd	0	2	intron			
Ppcdc	FBgn0038391	GATAe	1	0	upstream			
ppcs	FBgn0261703	gce	0	1	upstream	Met		Met (pubmed)
Ppcdc	FBgn0261703	gce	0	1	upstream			
Ppat-Dpck	FBgn0261703	gce	0	1	upstream			
ppcs	FBgn0004618	gl	0	1	upstream			
Dpck	FBgn0004618	gl	0	2	upstream			
ppcs	FBgn0259211	grh	1	0	upstream	Rel		
Ppat-Dpck	FBgn0259211	grh	1	0	upstream			
CG5828	FBgn0259211	grh	1	0	upstream			
Dpck	FBgn0001138	grn	0	1	upstream			

Ppat-Dpck	FBgn0001148	gsb	0	1	upstream		
ppcs	FBgn0001150	gt	0	1	upstream	ttk	
Dpck	FBgn0001150	gt	0	1	upstream		
CG5828	FBgn0001150	gt	1	2	upstream		
fbl	FBgn0001150	gt	0	1	intron	ttk	
Ppcdc	FBgn0001168	h	1	0	upstream		
Ppat-Dpck	FBgn0001168	h	1	0	upstream	dpn	ac, sc, l(1)sc (pubmed)
Dpck	FBgn0001168	h	0	1	upstream		
CG5828	FBgn0001168	h	2	0	upstream		ac, sc, l(1)sc (pubmed)
Ppat-Dpck	FBgn0032209	Hand	0	2	upstream		
fbl	FBgn0032209	Hand	0	1	intron		
ppcs	FBgn0001180	hb	0	13	upstream		Kr (pubmed)
ppcs	FBgn0001180	hb	0	5	intron		Kr (pubmed)
Ppcdc	FBgn0001180	hb	0	1	upstream		
Ppcdc	FBgn0001180	hb	0	3	intron		
Ppat-Dpck	FBgn0001180	hb	0	6	upstream		
Dpck	FBgn0001180	hb	0	16	upstream		
CG5828	FBgn0001180	hb	0	19	upstream		Kr (pubmed)
fbl	FBgn0001180	hb	0	3	upstream		
fbl	FBgn0001180	hb	0	3	intron		
fbl	FBgn0001185	her	0	1	intron		
Ppcdc	FBgn0027788	Hey	0	1	upstream		
Ppat-Dpck	FBgn0027788	Hey	0	1	upstream	dpn	
ppcs	FBgn0001204	hkb	0	2	intron		
Ppcdc	FBgn0001204	hkb	0	2	upstream		
Dpck	FBgn0001204	hkb	0	2	upstream		
fbl	FBgn0261283	HLH106	0	1	intron		
Ppat-Dpck	FBgn0011277	HLH4C	0	2	upstream		
Ppat-Dpck	FBgn0022740	HLH54F	0	3	upstream	da	
Ppcdc	FBgn0002609	HLHm3	0	2	upstream	h	
Ppat-Dpck	FBgn0002609	HLHm3	0	2	upstream	ac, sc, h	
Ppcdc	FBgn0002631	HLHm5	0	1	upstream	da	
Ppat-Dpck	FBgn0002631	HLHm5	0	3	upstream	da	
Ppat-Dpck	FBgn0002633	HLHm7	0	2	upstream	ac, sc, da	
ppcs	FBgn0002733	HLHmbeta	0	1	upstream	da, eg	
Ppcdc	FBgn0002733	HLHmbeta	0	1	upstream	da, tap	
Ppat-Dpck	FBgn0002733	HLHmbeta	0	1	upstream	sc, da	sc (pubmed)
Dpck	FBgn0002733	HLHmbeta	0	2	upstream		
CG5828	FBgn0002733	HLHmbeta	0	2	upstream	sc, da	sc (pubmed)
ppcs	FBgn0002734	HLHmd	0	2	upstream		
ppcs	FBgn0002734	HLHmd	0	1	intron		
Ppcdc	FBgn0002734	HLHmdelta	0	2	upstream		
Ppat-Dpck	FBgn0002734	HLHmdelta	0	3	upstream		
ppcs	FBgn0002735	HLHmg	0	2	upstream	da	
ppcs	FBgn0002735	HLHmg	0	1	intron	da	
Ppcdc	FBgn0002735	HLHmg	0	1	upstream	da	
Ppat-Dpck	FBgn0002735	HLHmgamma	0	3	upstream	sc, da, dpn, ac	

ppcs	FBgn0004914	Hnf4	0	1	upstream		
Ppcdc	FBgn0004914	Hnf4	0	1	upstream		
CG5828	FBgn0004914	Hnf4	0	1	upstream		
ppcs	FBgn0261239	Hr39	0	1	intron		EcR (pubmed)
ppcs	FBgn0000448	Hr46	0	2	upstream	toy	
ppcs	FBgn0000448	Hr46	0	2	intron	toy	
Ppcdc	FBgn0000448	Hr46	0	2	upstream	toy	
ppcs	FBgn0034012	Hr51	0	1	intron	Eip75B	
Ppat-Dpck	FBgn0034012	Hr51	0	1	upstream		
CG5828	FBgn0034012	Hr51	0	2	upstream		
fbl	FBgn0034012	Hr51	0	1	upstream	Eip75B	
ppcs	FBgn0015239	Hr78	0	1	upstream		
Ppcdc	FBgn0015239	Hr78	0	1	upstream		
Ppat-Dpck	FBgn0015239	Hr78	1	0	upstream		
CG5828	FBgn0015239	Hr78	1	1	upstream		
Dpck	FBgn0037436	Hr83	0	2	upstream	kni	
fbl	FBgn0037436	Hr83	0	1	intron		
Ppcdc	FBgn0001235	hth	1	0	upstream	ey	
Ppat-Dpck	FBgn0001235	hth	1	0	upstream	ey	
ppcs	FBgn0039350	jigr1	0	2	upstream		
Ppat-Dpck	FBgn0039350	jigr1	0	2	upstream		
Dpck	FBgn0039350	jigr1	0	2	upstream		
CG5828	FBgn0039350	jigr1	0	2	upstream		
fbl	FBgn0039350	jigr1	0	1	intron		
ppcs	FBgn0027339	jim	0	6	upstream		
Ppcdc	FBgn0027339	jim	0	1	upstream		
Ppat-Dpck	FBgn0027339	jim	0	3	upstream		
Dpck	FBgn0027339	jim	0	1	upstream		
CG5828	FBgn0027339	jim	0	15	upstream		
fbl	FBgn0027339	jim	0	4	upstream		
fbl	FBgn0001291	Jra	0	1	intron	kay	kay (pubmed)
ppcs	FBgn0001291	kay	0	3	upstream	Stat92E, kay	pnt, kay (pubmed)
ppcs	FBgn0001297	kay	0	3	upstream	vri	
Dpck	FBgn0001291	kay	0	1	upstream	kay	kay (pubmed)
Dpck	FBgn0001297	kay	0	1	upstream		
fbl	FBgn0001297	kay	0	1	intron	vri, Jra	Jra (pubmed)
ppcs	FBgn0011236	ken	0	2	upstream		
ppcs	FBgn0011236	ken	0	1	intron		
Ppcdc	FBgn0011236	ken	0	1	upstream		
CG5828	FBgn0011236	ken	0	1	upstream	Trl	
fbl	FBgn0011236	ken	0	2	upstream		
ppcs	FBgn0013469	klu	0	3	intron		
Ppcdc	FBgn0013469	klu	0	7	upstream		H (pubmed)
Dpck	FBgn0013469	klu	0	3	upstream		H (pubmed)
CG5828	FBgn0013469	klu	0	4	upstream		H (pubmed)
ppcs	FBgn0001320	kni	0	3	upstream		knrl (pubmed)
Dpck	FBgn0001320	kni	0	2	intron	Hr83	knrl (pubmed)
ppcs	FBgn0001323	knrl	0	3	upstream	CG5953	kni (pubmed)

Dpck	FBgn0001323	knrl	0	2	intron	CG5953		kni (pubmed)
ppcs	FBgn0001325	Kr	0	1	upstream			Eip75B, hb (pubmed)
CG5828	FBgn0001325	Kr	1	0	upstream			hb (pubmed)
Ppat-Dpck	FBgn0002561	l(1)sc	0	2	upstream	da		da, h (pubmed)
CG5828	FBgn0002561	l(1)sc	0	2	upstream	da		da, h (pubmed)
fbl	FBgn0002561	l(1)sc	0	1	upstream			
ppcs	FBgn0086910	l(3)neo38	0	1	upstream			
ppcs	FBgn0086910	l(3)neo38	0	1	intron			
Ppat-Dpck	FBgn0086910	l(3)neo38	0	1	upstream			
CG5828	FBgn0040918	Lag1	0	1	upstream			
ppcs	FBgn0039039	lmd	0	2	upstream			
CG5828	FBgn0039039	lmd	0	2	upstream			
Ppcdc	FBgn0005630	lola	0	1	upstream			
Dpck	FBgn0005630	lola-PJ	0	1	upstream			
Dpck	FBgn0040765	luna	0	2	upstream			
CG5828	FBgn0040765	luna	0	1	upstream			
ppcs	FBgn0017578	Max	0	1	upstream	Myc		Myc (pubmed)
Ppat-Dpck	FBgn0017578	Max	0	1	upstream	Myc		Myc (pubmed)
Dpck	FBgn0023215	Max	0	1	upstream	Max		Myc (pubmed)
fbl	FBgn0023215	Max	0	1	upstream	Max		Myc (pubmed)
ppcs	FBgn0011655	Med	1	0	upstream			
Ppcdc	FBgn0011655	Med	3	0	upstream			
Ppat-Dpck	FBgn0011655	Med	3	0	upstream			
Dpck	FBgn0011655	Med	3	0	upstream			
CG5828	FBgn0011655	Med	2	0	upstream			
ppcs	FBgn0037207	Mes2	0	2	upstream	CG12768		
fbl	FBgn0037207	Mes2	0	1	upstream			
fbl	FBgn0037207	Mes2	0	1	intron			
ppcs	FBgn0002723	Met	0	1	upstream	usp, EcR, gce		gce, br (pubmed)
ppcs	FBgn0023076	Met	0	2	upstream	cyc		
ppcs	FBgn0002723	Met	0	1	intron	usp, EcR, gce		gce, br (pubmed)
ppcs	FBgn0023076	Met	0	3	intron	cyc		
ppcs	FBgn0032940	Mio	0	1	upstream			
ppcs	FBgn0039509	Mio	0	1	upstream			
Ppat-Dpck	FBgn0032940	Mio	0	1	upstream			
Ppat-Dpck	FBgn0039509	Mio	0	1	upstream			
ppcs	FBgn0262656	Myc	2	1	upstream	Max, Ets97D		Max (pubmed)
Ppcdc	FBgn0262656	Myc	1	0	upstream			
Ppat-Dpck	FBgn0262656	Myc	0	1	upstream	Max		Max (pubmed)
Dpck	FBgn0262656	Myc	2	0	upstream	Max		prd, Max (pubmed)
CG5828	FBgn0262656	Myc	1	0	upstream	Ets97D		
fbl	FBgn0262656	Myc	1	0	upstream	Max		Max (pubmed)
Ppcdc	FBgn0002922	nau	0	2	upstream			
ppcs	FBgn0030505	NFAT	0	2	intron			pnr (pubmed)
ppcs	FBgn0085424	nub	0	1	upstream	pdm2		
ppcs	FBgn0085424	nub	0	11	intron	pdm2		
ppcs	FBgn0002985	odd	0	1	upstream			
fbl	FBgn0032651	Oli	0	1	upstream			

ppcs	FBgn0003002	Opa	0	1	upstream	croc	
Ppat-Dpck	FBgn0003028	ovo	0	1	upstream		
ppcs	FBgn0004394	pdm2	0	6	intron	nub	
Ppcdc	FBgn0016694	Pdp1	0	6	upstream		
Dpck	FBgn0016694	Pdp1	0	2	upstream		
Dpck	FBgn0003053	peb-F5-7	0	1	upstream		
fbl	FBgn0003053	peb-F5-7	0	1	intron		Jra (pubmed)
Ppcdc	FBgn0002521	pho	0	1	upstream	Sp1	
Dpck	FBgn0002521	pho	0	4	upstream	Sp1	
CG5828	FBgn0002521	pho	0	1	upstream	Sp1	
fbl	FBgn0002521	pho	0	1	intron	Sp1	
ppcs	FBgn0035997	phol	0	1	intron		
Ppcdc	FBgn0035997	phol	0	2	upstream		
Dpck	FBgn0035997	phol	0	6	upstream		
fbl	FBgn0035997	phol	0	2	upstream		
ppcs	FBgn0003117	pnr	0	1	upstream	tin, tup	NFAT (pubmed)
CG5828	FBgn0003117	pnr	0	1	upstream	tup	
fbl	FBgn0003117	pnr	0	3	intron		
ppcs	FBgn0003118	pnt	0	2	upstream		aop (pubmed)
CG5828	FBgn0003118	pnt	0	2	upstream		aop (pubmed)
Dpck	FBgn0003145	prd	1	0	upstream	bin, croc	Myc (pubmed)
ppcs	FBgn0014018	Rel	0	2	intron	br, grh, sqz	
ppcs	FBgn0004795	retn	0	2	upstream		
ppcs	FBgn0004795	retn	0	2	intron		
Ppcdc	FBgn0004795	retn	0	2	upstream		
ppcs	FBgn0003254	rib	0	1	upstream	ttk, br	
ppcs	FBgn0003254	rib	0	1	intron	ttk, br	
Ppat-Dpck	FBgn0003254	rib	0	2	upstream	br	
Dpck	FBgn0003254	rib	0	2	upstream	br	
CG5828	FBgn0003254	rib	0	3	upstream	br	
fbl	FBgn0003254	rib	0	2	upstream	ttk, br	
fbl	FBgn0003254	rib	0	1	intron	ttk, br	
ppcs	FBgn0259172	rn	0	2	upstream		
Ppat-Dpck	FBgn0259172	rn	0	6	upstream		
Dpck	FBgn0259172	rn	0	2	upstream		
CG5828	FBgn0259172	rn	0	13	upstream		
ppcs	FBgn0003300	run	0	1	upstream		ttk (pubmed)
ppcs	FBgn0013753	run	0	1	upstream	run	
ppcs	FBgn0003300	run	0	1	intron		ttk (pubmed)
ppcs	FBgn0013753	run	0	1	intron	run	
Ppcdc	FBgn0003300	run	0	1	upstream	H	
Ppcdc	FBgn0013753	run	0	1	upstream	run	
Dpck	FBgn0003300	run	0	1	upstream	H	
Dpck	FBgn0013753	run	0	1	upstream	run	
fbl	FBgn0003300	run	0	1	intron		ttk (pubmed)
fbl	FBgn0013753	run	0	1	intron	run	
Ppcdc	FBgn0037672	sage	0	2	upstream		
Ppat-Dpck	FBgn0000287	salr-F3-5	0	2	upstream		

Dpck	FBgn0000287	salr-F3-5	0	2	upstream		
Ppat-Dpck	FBgn0004170	sc	0	2	upstream	da	ac, da, h (pubmed)
CG5828	FBgn0004170	sc	0	2	upstream	da	ac, da, tup, h (pubmed)
fbl	FBgn0004170	sc	0	1	upstream		ac (pubmed)
ppcs	FBgn0002573	sens	0	1	upstream	CG5953	
Ppcdc	FBgn0002573	sens	0	1	upstream	CG5953	Dl (pubmed)
ppcs	FBgn0051632	sens2	0	1	upstream		
Ppcdc	FBgn0051632	sens2	0	1	upstream		
ppcs	FBgn0003396	shn-F1-2	0	1	intron		
Ppcdc	FBgn0003396	shn-F1-2	0	1	upstream		
Ppcdc	FBgn0032741	Side	0	2	upstream		
ppcs	FBgn0005638	slbo	0	1	upstream		Stat92E (pubmed)
Ppat-Dpck	FBgn0005638	slbo	0	1	upstream		
fbl	FBgn0005638	slbo	0	2	upstream		
ppcs	FBgn0003430	slp1	0	2	upstream		
ppcs	FBgn0003430	slp1	0	1	intron		
Ppcdc	FBgn0003430	slp1	0	1	upstream		
Ppat-Dpck	FBgn0003430	slp1	0	1	upstream		
fbl	FBgn0003430	slp1	0	5	intron		
fbl	FBgn0003430	slp1	0	1	upstream		
ppcs	FBgn0004567	slp2	0	2	upstream		
ppcs	FBgn0004567	slp2	0	1	intron		
Ppcdc	FBgn0004567	slp2	0	1	upstream		
Ppat-Dpck	FBgn0004567	slp2	0	1	upstream		
fbl	FBgn0004567	slp2	0	6	intron		
fbl	FBgn0004567	slp2	0	2	upstream		
ppcs	FBgn0004892	sob	0	1	upstream		
ppcs	FBgn0005612	Sox14	0	1	intron		
Ppat-Dpck	FBgn0005612	Sox14	0	2	upstream		
ppcs	FBgn0020378	Sp1	0	1	upstream		
Ppcdc	FBgn0020378	Sp1	0	1	upstream	pho	
Dpck	FBgn0020378	Sp1	0	6	upstream	pho	
CG5828	FBgn0020378	Sp1	0	3	upstream	pho	
fbl	FBgn0020378	Sp1	0	3	upstream	pho	
ppcs	FBgn0010768	sqz	0	25	upstream	Rel	
ppcs	FBgn0010768	sqz	0	20	intron	Rel	
Ppcdc	FBgn0010768	sqz	0	1	intron	dimm	
Ppat-Dpck	FBgn0010768	sqz	0	9	upstream		
Dpck	FBgn0010768	sqz	0	23	upstream	dimm	
CG5828	FBgn0010768	sqz	0	56	upstream		
fbl	FBgn0010768	sqz	0	7	upstream		
fbl	FBgn0010768	sqz	0	2	intron		
ppcs	FBgn0003499	sr	0	1	intron		
Ppcdc	FBgn0003499	sr	0	6	upstream		
ppcs	FBgn0003507	srp	0	1	upstream		
Ppcdc	FBgn0003507	srp	0	2	upstream		
Ppat-Dpck	FBgn0003507	srp	0	1	upstream		
Dpck	FBgn0003507	srp	0	1	upstream		

fbl	FBgn0003507	srp	0	1	upstream		
fbl	FBgn0003507	srp	0	1	intron		
ppcs	FBgn0016917	STAT92E	0	2	intron	toy, ttk	slbo (pubmed)
ppcs	FBgn0033782	sug	0	1	upstream		
Dpck	FBgn0033782	sug	0	1	upstream		
CG5828	FBgn0033782	sug	0	1	upstream		
ppcs	FBgn0003651	svp	0	1	upstream		
Ppcdc	FBgn0003651	svp	0	1	upstream		
CG5828	FBgn0003651	svp	0	1	upstream		
ppcs	FBgn0041092	tai	0	1	upstream	usp, ab, EcR	
ppcs	FBgn0041092	tai	0	2	intron	usp, ab, EcR	
Ppcdc	FBgn0023076	tai	0	1	upstream	cyc	
Ppcdc	FBgn0041092	tai	0	1	upstream	usp, ab	
Ppcdc	FBgn0015550	tap	0	2	upstream		
ppcs	FBgn0264075	tgo	1	0	intron		
ppcs	FBgn0004666	tgo	0	4	upstream	tgo	tgo (pubmed)
ppcs	FBgn0015014	tgo	0	10	upstream		
ppcs	FBgn0015542	tgo	0	4	upstream		
ppcs	FBgn0262139	tgo	0	2	upstream	tgo	tgo (pubmed)
Ppat-Dpck	FBgn0264075	tgo	1	0	upstream		
Ppat-Dpck	FBgn0003513	tgo	0	1	upstream	tgo	Dll, tgo (pubmed)
Ppat-Dpck	FBgn0015014	tgo	0	3	upstream		
Dpck	FBgn0004666	tgo	0	2	upstream	D, tgo	tgo (pubmed)
Dpck	FBgn0015014	tgo	0	1	upstream		
fbl	FBgn0015014	tgo	0	1	upstream		
ppcs	FBgn0004110	tin	0	1	intron	pnr	
Ppcdc	FBgn0004110	tin	0	1	upstream		
Ppcdc	FBgn0000964	tj	0	2	upstream		
CG5828	FBgn0000964	tj	0	1	upstream		
Dpck	FBgn0003720	tll	0	1	upstream		
fbl	FBgn0003720	tll	0	1	upstream		
ppcs	FBgn0019650	toy	0	2	intron	Stat92E	ey (pubmed)
Ppcdc	FBgn0019650	toy	0	2	upstream		ey (pubmed)
Ppat-Dpck	FBgn0013263	Trl	1	0	upstream		
CG5828	FBgn0013263	Trl	1	0	upstream	CG12155, ken	
ppcs	FBgn0003870	ttk	0	1	intron	gt, Stat92E, rib	run, aop (pubmed)
fbl	FBgn0003870	ttk	0	3	upstream	gt, D, rib	ac, run (pubmed)
ppcs	FBgn0003896	tup	0	1	upstream	pnr	
Ppcdc	FBgn0003896	tup	0	2	upstream		
CG5828	FBgn0003896	tup	0	2	upstream	pnr	sc (pubmed)
ppcs	FBgn0029711	Usf	0	1	intron		
ppcs	FBgn0003964	usp	0	1	upstream	Met, tai, EcR	
Ppcdc	FBgn0003964	usp	0	1	intron	tai	
Ppat-Dpck	FBgn0003964	usp	0	1	upstream	EcR	
ppcs	FBgn0003986	Vnd	0	2	intron		
Ppcdc	FBgn0003986	Vnd	0	1	intron		
fbl	FBgn0003986	Vnd	0	1	upstream		
ppcs	FBgn0016076	vri	0	2	upstream	kay	

fbl	FBgn0016076	vri	0	2	upstream	kay	
ppcs	FBgn0021872	Xbp1	0	1	upstream		
ppcs	FBgn0261113	Xrp1	0	1	upstream		
Ppat-Dpck	FBgn0036126	Xrp1	0	1	upstream	Xrp1, crc	
Ppat-Dpck	FBgn0261113	Xrp1	0	1	upstream		
fbl	FBgn0036126	Xrp1	0	4	upstream	Xrp1	
fbl	FBgn0261113	Xrp1	0	4	upstream		
ppcs	FBgn0004050	z	0	1	intron		
Ppat-Dpck	FBgn0004050	z	1	0	upstream		
fbl	FBgn0004050	z	0	1	upstream		
Ppcdc	FBgn0004053	zen	0	1	upstream		
Ppat-Dpck	FBgn0004606	zfh1	0	1	upstream		
CG5828	FBgn0004606	zfh1	1	0	upstream		
fbl	FBgn0004606	zfh1	0	1	upstream		
fbl	FBgn0004606	zfh1	0	1	intron		
ppcs	FBgn0259789	zld	2	1	intron		
ppcs	FBgn0259789	zld	1	1	upstream		
Ppat-Dpck	FBgn0259789	zld	1	0	upstream		
Dpck	FBgn0259789	zld	1	0	upstream		
CG5828	FBgn0259789	zld	1	0	upstream		
fbl	FBgn0259789	zld	1	0	upstream		

Supplementary table3. Genotypes used in this study

Figure	
1, 2c, d, h, S4b	*w1118*
	CG31272 (MT)-GAl4, Tub-GAL80TS > +
2e, f, g, l, j, k; 3a, c, d; S1e, f, g, j; S2a, b c, d, f, g	*CG31272-GAl4, Tub-GAL80TS > CG5828 (dPANK4)-RNAi*
	esg-GAL4, tub-GAL80TS > +
S1b	*esg-GAL4, tub-GAL80TS > Fbl-RNAi*
	CG31272-GAl4, Tub-GAL80TS > +
S1h, i	*CG31272-GAl4, Tub-GAL80TS > CG5828*
	esg-GAL4, tub-GAL80TS > +
	esg-GAL4, tub-GAL80TS > Hmgcr-RNAi
	esg-GAL4, tub-GAL80TS > Qm-RNAi
3e	*esg-GAL4, tub-GAL80TS > beta GGT-I-RNAi*
	CG31272-GAl4, Tub-GAL80TS > +
3f	*CG31272-GAl4, Tub-GAL80TS > Drip-RNAi*
	esg-GAL4, tub-GAL80TS > +
	esg-GAL4, tub-GAL80TS > Mof-RNAi
	esg-GAL4, tub-GAL80TS > Hat1-RNAi
S2e	*esg-GAL4, tub-GAL80TS > Gcn5-RNAi*
	tsh-GAl4, Tub-GAL80TS > +
S2i	*tsh-GAl4, Tub-GAL80TS > Drip-RNAi*
	esg-GAL4, tub-GAL80TS > UAS-GFP
4a, 5f	*esg-GAL4, tub-GAL80TS > UAS-GFP, UAS-yki3SA*
	esg- LexA, tub-GAL80TS > +; CG31272>+
	esg- LexA, tub-GAL80TS > LexAop-yki3SA-GFP 2nd; CG31272>+
4b	*esg- LexA, tub-GAL80TS > LexAop-yki3SA-GFP 2nd; CG31272>Smvt-RNAi*
	esg- LexA, tub-GAL80TS > +; CG31272>+
	esg- LexA, tub-GAL80TS > LexAop-yki3SA-GFP 2nd; CG31272>+
	esg- LexA, tub-GAL80TS > LexAop-yki3SA-GFP 2nd; CG31272>Fbl-RNAi
4c, d, e, f, g, l, j, k, l, m; S3b, c, d, e, m, l	*esg- LexA, tub-GAL80TS > LexAop-yki3SA-GFP 2nd; CG31272>CG5828*
S3f, g, h, l, j, k	*esg- LexA, tub-GAL80TS > +; CG31272>+*
	esg- LexA, tub-GAL80TS > LexAop-yki3SA-GFP 3rd; CG31272>+
	esg- LexA, tub-GAL80TS > LexAop-yki3SA-GFP 3rd; CG31272>Fbl-RNAi
	esg- LexA, tub-GAL80TS > LexAop-yki3SA-GFP 3rd; CG31272>CG5828
	CG31272-GAl4, Tub-GAL80TS > +
5a	*CG31272-GAl4, Tub-GAL80TS > Myc*
5c	*CG31272-GAl4, Tub-GAL80TS > Myc-HA*
	CG31272-GAl4, Tub-GAL80TS > +
S4c, d	*CG31272-GAl4, Tub-GAL80TS > Myc-RNAi*
	esg- LexA, tub-GAL80TS > +; CG31272>+
	esg- LexA, tub-GAL80TS > LexAop-yki3SA-GFP 2nd; CG31272>+
5g, h, l, j, k, l, m n , S4e, f, g, h	*esg- LexA, tub-GAL80TS > LexAop-yki3SA-GFP 2nd; CG31272>Myc-RNAi*
	esg- LexA, tub-GAL80TS > +; CG31272>+
	esg- LexA, tub-GAL80TS > LexAop-yki3SA-GFP 3rd; CG31272>+
S4i, j, k, l, m, n	*esg- LexA, tub-GAL80TS > LexAop-yki3SA-GFP 3rd; CG31272>Myc-RNAi*
	CG31272-GAl4, Tub-GAL80TS > +
5o	*CG31272-GAl4, Tub-GAL80TS > PvR[Act]*
5p	*esg- LexA, tub-GAL80TS > +; CG31272>+*

	esg- LexA, tub-GAL80TS > LexAop-yki3SA-GFP 2nd; CG31272>+
	esg- LexA, tub-GAL80TS > LexAop-yki3SA-GFP 2nd; CG31272>Pvr-RNAi

Supplementary table4. Primers used in this study

	**RT-qPCR primers**
dPANK4 (CG5828)	TTACAGATCCCTGGCTGAGAC
	CCACCTTGTGTCCTCATCCG
Fbl	TTCTCTTCGCCGATCTGCATA
	GAACTGCTGCTTTTCGCTTTTTA
FASN1	GACATGGTCAACGATGATCCC
	ACCGAAGAACTGTTGGTCAAAG
ACC	ACAAGATGAAGAACCATGCCAT
	TTCGCGGGACTTCTGTTGC
AcCoAS	CCATGATTCTGGAGCTGCCTA
	GCCTTCAGGTACAGGGGTTTC
Atpcl	TTTCCACAGTAAATTCCACGACA
	GGCGCTTGATAAGTTGATCGG
Gcn5	GGTGGAAACAAGAGGACCAGTG
	CCAAATTCTCACTGCTTGGA
Elp3	AATTCTGCTTCCAAAGCTGAGG
	GCCGGGACAATAGACGCATA
Hat1	TGGTAGACTTTAAGCTGATCCGT
	CTCCCCGAAAATCTGGTGGG
Mof	GAGCCAACCGATGCGTACA
	TCCTCCGAAATGGGACTGATG
Nej	ATGATGGCCGATCACTTAGACG
	GATTTGTGGTTACACCGGAGG
Fpps	GCAACGCCTGATCTCTACCAG
	TTGGAGCGTCGATAAGGTTCT
Hmgcr	GCTGCACTGCCGTACTGTA
	AATGCCCAGCACATATTTGGA
Qm	TAAATGCGGCCAACTATGCAC
	CATCAGCTTGTAATCCGACTCG
beta GGT-I	ATGGCCTCGCACGATAACAC
	GCAATGAGTTTAGCACATCCAGG
CG13200	GCATATGCGACAAAGTGGGCC
	AACATTCACCGCAAGGGCTCC
RP49	AAGAAGCGCACCAAGCACTTCATC
	TCTGTTGTCGATACCCTTGGGCTT
	**ChIP-qPCR primers**
dPANK4 (CG5828)	CCAGCTGAGGTGTGCTGG
	TTGAACAGTCTTATTGCAACTATCG
Fbl	GGTGCACATAAAATGTGTGGGA
	ATCGAAAAGCGCAGTGTTGG
Tll (Neg)	CCTTCTTGAATTTCCAGGTCGC
	CGTCTTGTCCACCACACAGA
